# Equilibrium-point control of human elbow-joint movement under isometric environment by using multichannel functional electrical stimulation

**DOI:** 10.3389/fnins.2014.00164

**Published:** 2014-06-17

**Authors:** Kazuhiro Matsui, Yasuo Hishii, Kazuya Maegaki, Yuto Yamashita, Mitsunori Uemura, Hiroaki Hirai, Fumio Miyazaki

**Affiliations:** ^1^Department of Systems Science, Faculty of Engineering Science, Osaka UniversityOsaka, Japan; ^2^Fujitsu LimitedKanagawa, Japan

**Keywords:** functional electrical stimulation (FES), equilibrium-point control, EAA ratio, EAA activity, muscle synergy

## Abstract

Functional electrical stimulation (FES) is considered an effective technique for aiding quadriplegic persons. However, the human musculoskeletal system has highly non-linearity and redundancy. It is thus difficult to stably and accurately control limbs using FES. In this paper, we propose a simple FES method that is consistent with the motion-control mechanism observed in humans. We focus on joint motion by a pair of agonist-antagonist muscles of the musculoskeletal system, and define the “electrical agonist-antagonist muscle ratio (EAA ratio)” and “electrical agonist-antagonist muscle activity (EAA activity)” in light of the agonist-antagonist muscle ratio and agonist-antagonist muscle activity, respectively, to extract the equilibrium point and joint stiffness from electromyography (EMG) signals. These notions, the agonist-antagonist muscle ratio and agonist-antagonist muscle activity, are based on the hypothesis that the equilibrium point and stiffness of the agonist-antagonist motion system are controlled by the central nervous system. We derived the transfer function between the input EAA ratio and force output of the end-point. We performed some experiments in an isometric environment using six subjects. This transfer-function model is expressed as a cascade-coupled dead time element and a second-order system. High-speed, high-precision, smooth control of the hand force were achieved through the agonist-antagonist muscle stimulation pattern determined by this transfer function model.

## 1. Introduction

In recent years, the number of people affected by strokes and spinal cord injuries has increased because the rapidly aging population and the high incidence of traffic accidents in automobilized societies. Many studies have been conducted on movement support and functional compensation for paralyzed individuals. The use of functional electrical stimulation (FES) to induce muscle activity via direct electrical stimulation of peripheral muscles has attracted particular attention. FES has even been used to assist severely paralyzed patients. According to reported adaptation examples (Giuffrida et al., 2001; Widjaja et al., 2011), FES can help with spastic paralysis in stroke patients. Muscle stimulation is performed by refereing to the antagonistic muscle’s electromyogram (EMG). FES can also be used for treating tremor paralysis patients. In this approach, muscle stimulation is performed by refereing to limb tremor. Furthermore, many studies focused on joint trajectory tracking by electrical stimulation of multiple muscles have been reported. They are classified as open-loop (Bernotas et al., 1987; Buckett et al., 1987; Hoshimiya et al., 1989; Miller et al., 1989; Chizeck et al., 1991; Veltink et al., 1992; Smith et al., 1996; Chen et al., 1997; Davoodi et al., 1998; Rakos et al., 1999; Ferrarin et al., 2001; Watanabe et al., 2002a,b), closed-loop (Chizeck et al., 1980; Crago et al., 1980; Wilhere et al., 1985; Lemay et al., 1997), and hybrid type (Lan et al., 1994; Abbas et al., 1995; Kostov et al., 1995; Chang et al., 1997; Jonic et al., 1999; Qi et al., 1999; Adamczyk et al., 2000; Sites et al., 2000; Ianno et al., 2002; Kurosawa et al., 2005) applications. The hybrid type use of FES shows promise as a control method that combines the advantages of feedforward control, which allows for quick movement without delay, and feedback control, which reduces the effects of disturbance due to fatigue and load. However, it is difficult to derive an appropriate model for inclusion in the controller, because (1) the electrical stimulated musculoskeletal system is characterized by high non-linearity between stimulus current values and muscle force/length and (2) the control of joints that are moved by agonist-antagonistic muscle pairs is an ill-posed problem (Kurosawa et al., 2005), because of the redundancy in joint motion control.

In the field of exercise physiology, the equilibrium point hypothesis states that the stiffness and equilibrium point of the agonist-antagonist drive system are controlled by the central nervous system (Feldman, 1986). In addition, it has been shown that the muscle agonist-antagonist ratio is closely related to the joint angle corresponding to the equilibrium point, and muscle agonist-antagonist activity has a close relationship with the joint stiffness, as is evident from the results of analyses of muscle agonist-antagonist ratio and muscle agonist-antagonist activity (Iimura et al., 2011; Ariga et al., 2012). The muscle agonist-antagonist ratio is represented by the ratio of the EMGs of agonist-antagonistic muscle pair groups, which make up the musculoskeletal system. The muscle agonist-antagonist activity is represented by the sum of the agonist-antagonistic muscle pair group’s EMGs. The equilibrium point and joint stiffness can be determined independently based on muscle agonist-antagonist ratio and activity. The muscle agonist-antagonist ratio and activity are used to control multiple pneumatic artificial muscles (Pham et al., 2014). The concept of muscle agonist-antagonist ratio or activity can be useful in electrically stimulating the muscle pair group as well.

In this study, we focused on non-linearity and redundancy in developing a method for applying the concept of the muscle agonist-antagonist ratio and activity to electrical stimulation. Problems such as non-linearity and redundancy are encountered when FES is used for controlling the human body. The concept of the muscle agonist-antagonist ratio or activity can be used to determine the equilibrium point and joint stiffness, which are considered in the equilibrium point hypothesis. We assume that we can linearly approximate human motion control by determining the equilibrium point and joint stiffness and by controlling the equilibrium point independently with the help of the EAA ratio and activity, which are based on the concept of the muscle agonist-antagonist ratio and activity. As an example, we use the human elbow joint, which is an antagonistic drive system. We attempt to model the human elbow joint using the proposed method and use the modeling results to control the end-point force (hand force) in an isometric environment. In addition, we conducted experiments to assess the trajectory tracking performance achieved with the method developed.

## 2. Materials and methods

### 2.1. Experimental environment

**Figure 1:**
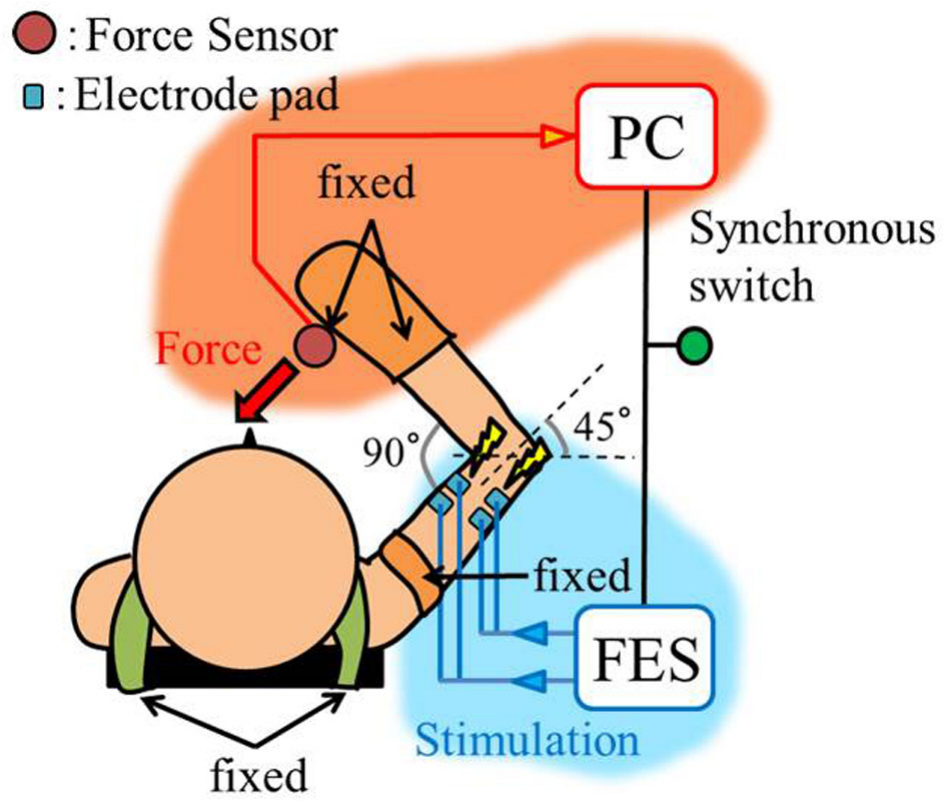
**Experimental setup, top view**.

**Figure 2:**
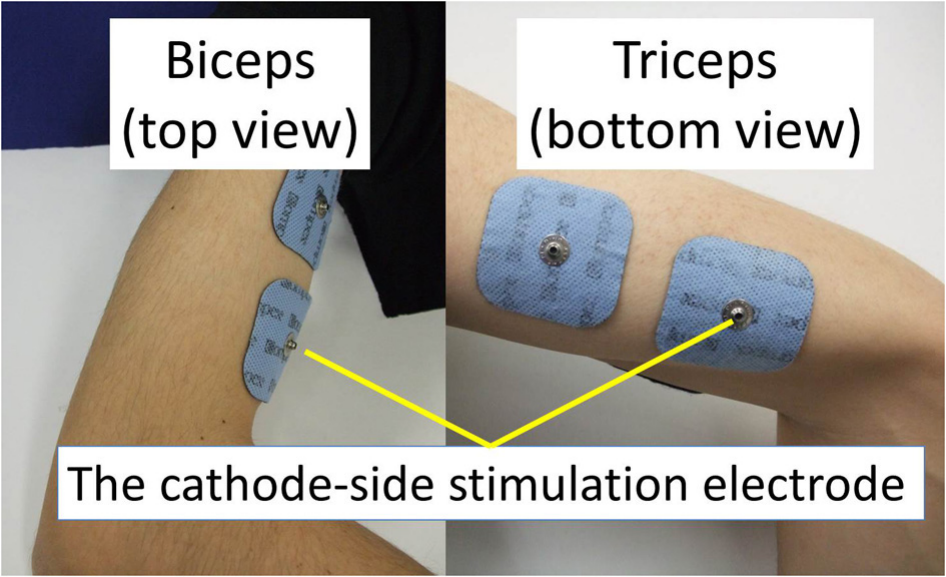
**Stimulation electrodes installed**.

The experimental environment and system configuration are shown in Figure 1. A stimulator manufactured by Multi Channel Systems, Inc. (STG4008) is used for electrically stimulation of the target muscles. The STG4008 can control the stimulus current value. Based on the results of attempts to use various modulation schemes, a sinusoidal electrical stimulation pattern with a frequency of 60 (Hz), generated using the AM (Amplitude Modulation) method, was chosen because it yielded the greatest effect and resulted in the least discomfort. We control only the amplitude of the sine-wave, with the base frequency fixed at 60 (Hz). The cathode-side stimulation electrode is installed at a motor point in the stimulated target muscles, which are the biceps and triceps of the subject’s right upper arm (Figure 2). A stimulation electrode made by Compex Inc. (Electrode for performance/energy) is used. The motor-points are searched using motor-point pen made by Compex Inc. Before we apply the electrodes, we apply electrode gel made by Compex Inc. to the skin to decrease impedance. During the procedure, the right upper arm is held in a horizontal plane by the seat, the wrist is secured with splint material, and the trunk is fixed to the chair with a shoulder belt. The hand force is sampled at a rate of 1000 (Hz) using a three-axis force sensor made by Tech-Gihan, Inc. (USL06-H5-200N). Negative measurements denote flexion and positive measurements denote extension. The experiment is conducted in an isometric environment, and the angle between the upper arm and the body surface is 45°, while the elbow angle is maintained at 90°. Healthy adult males A (aged 27 years, right-handed), B (aged 24 years, right-handed), C (aged 21 years, right-handed), D (aged 24 years, right-handed), E (aged 24 years, right-handed), and F (aged 24 years, right-handed) volunteered to participate in the experiment. To eliminate the influence of fatigue, the experiments were limited to 1 min in duration. The purpose and the details of the experiment were explained to the subjects, and they agreed to participate in the experiment. The experiments were conducted with the approval of the Osaka University of Engineering Science Ethics Committee and in accordance with their prescribed procedures.

### 2.2. Electrical agonist-antagonist muscle ratio (EAA ratio) and the equilibrium point

We define the elbow joint as the control target. We focus on coordination between the triceps and the biceps. The triceps and biceps act during extension and flexion, respectively, of the elbow joint. We intend to simultaneously stimulate these two muscles. To this end, it is important to understand how humans generate this movement. Human muscle groups have multiple degrees of freedom, and humans operate various muscle groups simultaneously when generating a movement. The human body has different types of solutions that control the various body movements. This implies that any of the solution can be involved in the human body movement. For example, EMG analysis is performed to determine humans’ primary motion control. EMG presents a command signal to the muscle from the central nervous system. In this study, we focused on the EMG analysis method. The method is based on a combination of agonist-antagonist muscles. Iimura et al. defined *m*_*f*_ and *m*_*e*_ as the degrees of flexor and extensor muscle activity, respectively, of agonist-antagonist muscle pairs obtained from EMG. The agonist-antagonist muscle ratio *r* and the agonist-antagonist muscle activity *a* are given by Equations (1, 2), respectively. Iimura et al. showed that both *r* and *a* contribute to the joint equilibrium point and joint stiffness.

(1) $$r=\frac{m_{e}}{m_{f}+m_{e}}$$

(2) $$a=m_{f}+m_{e}$$

Electrical stimulation contracts human muscles. In this study, the normalized FES intensity to the biceps and triceps are defined as *I*_*f*_ (−) and *I*_*e*_ (−), respectively, and the electrical agonist-antagonist muscle ratio (EAA ratio) *r*_*E*_ and electrical muscle activity *a*_*E*_, which are obtained using Equations (1, 2) are defined as the new control variables.

(3) $$r_{E}=\frac{I_{e}}{I_{f}+I_{e}}$$

(4) $$a_{E}=I_{f}+I_{e}$$

Note that to minimize differences in the characteristics of the flexor and extensor and facilitate the extraction of the transfer characteristics, the stimulus current values are normalized. The maximum stimulus current *I*′_*fmax*_ (mA) and current *I*′_*emax*_ (mA) at which the subject does not feel pain, and the minimum stimulus current *I*′_*fmin*_ (mA) and current *I*′_*emin*_ (mA) at which muscle contraction commences are used for normalization, as shown bellow:

(5) $$I_{f}=({I^{\prime }}_{f}-{I^{\prime }}_{fmin})/({I^{\prime }}_{fmax}-{I^{\prime }}_{fmin})$$

(6) $$I_{e}=({I^{\prime }}_{e}-{I^{\prime }}_{emin})/({I^{\prime }}_{emax}-{I^{\prime }}_{emin})$$

Where, *I*′_*f*_, *I*′_*e*_ are the stimulus current values. If *r*_*E*_ is considered to contribute to the joint equilibrium point in a manner similar to that in EMG analysis, any change in *r*_*E*_ appears as a change in the hand force under constraints on hand movement, i.e., in an isometric environment. In this study, we investigate hand force in an isometric environment, as *r*_*E*_ is changed while *a*_*E*_ remains constant.

### 2.3. Electrical agonist-antagonist muscle activity (EAA activity) and the joint stiffness

**Figure 3:**
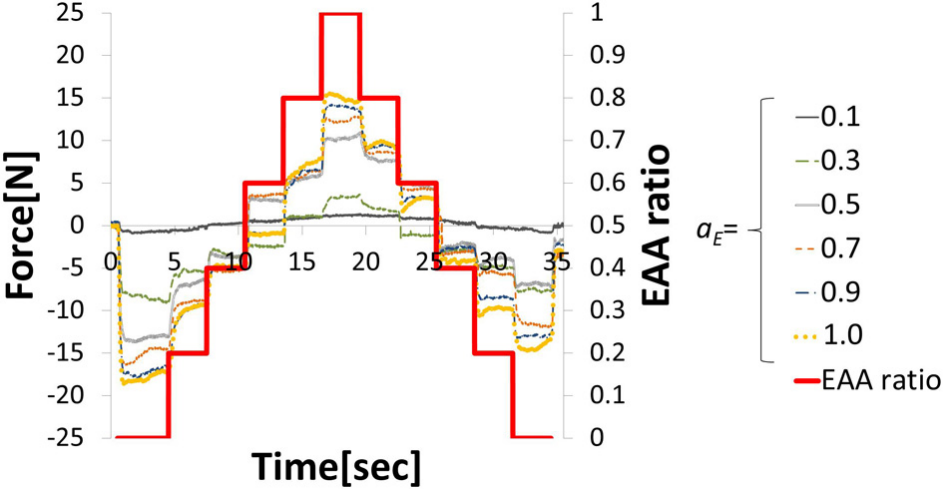
**Hand force for various levels of EAA activity**.

In EMG analysis, how muscle activity *a* contributes to joint stiffness has been shown by Iimura et al. (2011. To confirm that EAA activity *a*_*E*_ contributes to the joint stiffness in the same way, we conducted an experiment to increase or decrease the EAA ratio *r*_*E*_ from *r*_*E*_ = 0 to 1.0 in increments of 0.2 every 3 s (*a*_*E*_ = {0.1, 0.3, 0.5, 0.7, 0.9, 1.0}). The averages of three trials for each *a*_*E*_ are shown along with EAA input ratio in Figure 3. The results confirm that the displacement of the hand force increases with *a*_*E*_. In addition, when we estimated the transfer function(discussed more below), we determined values of the natural angular frequency ω_*n*_ for three values of *a*_*E*_ = {0.5, 0.8, 1.0} for subject A. We found that for *a*_*E*_ = 1.0, ω_*n*_ = 20.5 (rad/s); for *a*_*E*_ = 0.8, ω_*n*_ = 19.0 (rad/s); and for *a*_*E*_ = 0.5, ω_*n*_ = 14.3 (rad/s). These findings indicate that *a*_*E*_ contributes to the joint stiffness.

### 2.4. Control model

**Figure 4:**
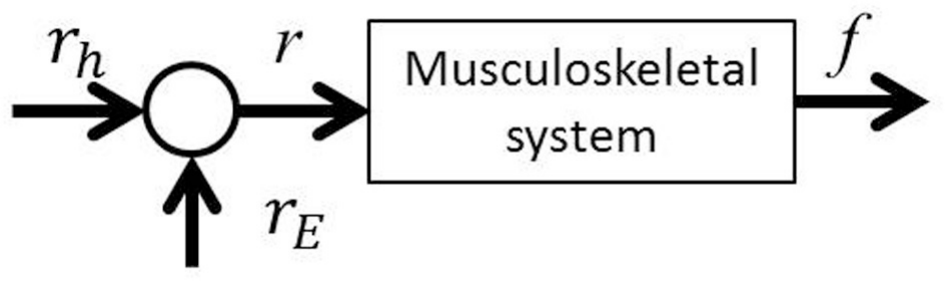
**EAA ratio-based FES control scheme**.

If we consider motion control of the elbow joint from the perspective of the equilibrium point hypothesis, it is possible to define two parameters as the control variables: joint stiffness and equilibrium point. In this paper, we report on a method for controlling the elbow joint using the EAA ratio: the equilibrium point. As Figure 4 shows, the exercise command *r*_*E*_ from the external FES current to the muscle groups is added to the list of movement commands *r*_*h*_ from the central nervous system to the agonist-antagonist muscle groups (the agonist-antagonist muscle ratio). Agonist-antagonist muscle pairs are driven by the movement command *r* (= *r*_*h*_ + *r*_*E*_), and as a result, hand force *f* is generated in an isometric environment. To confirm this theory, a constant value of EAA activity *a*_*E*_ = 1.0 was used.

### 2.5. Modeling

#### 2.5.1. Input–output of elbow joint system

In this study, to achieve elbow joint control using the EAA ratio, we experimentally determined the frequency characteristics between the hand force and the electrical stimulation input to the elbow joint system so that we could determine the transfer function for which the input is the EAA ratio and the output is the hand force in the isometric environment. Given that we seek the transfer function of the elbow joint system, a sine-wave EAA ratio with various periods *T* (s) was input to the muscles and the steady-state hand force was measured. We then used one cycle of one sine-wave input, perform sin-cos approximation using a multiple regression model, and expressed the result as a sine-wave. Thus, we obtained the output amplitude and phase of each period, as well as the corresponding frequency characteristics of the values obtained. The EAA ratio can be expressed as a function of time as follows.

(7) $$r_{E}(t)=-0.5sin\text{ ​}\left(\frac{2\pi }{T}t\right)\;+\;0.5$$

For the input, the EAA ratio was set to a sine-wave with possible values from 0 to 1, and the stimulation current value was determined. The normalized stimulation currents of each muscle, *I*_*e*_(*t*), *I*_*f*_(*t*), determined using Equations (3, 4), were calculated from the fixed electrical muscle activity *a*_*E*_, and the EAA ratio *r*_*E*_ was determined using Equation (7). The stimulus current values *I*′_*e*_(*t*), *I*′_*f*_(*t*), that were actually applied to the muscle, were determined from the maximum stimulation amplitude *I*′_*max*_ (mA), and minimum stimulus amplitude *I*′_*min*_ (mA) determined in advance. *I*′_*max*_ and *I*′_*min*_ are shown in Table 1. The resulting, *I*′_*e*_(*t*) and *I*′_*f*_(*t*) were determined from Equations (8, 9) in the case of subject B, for example. The hand force *f*(*t*) that appears as an output is approximated using the multiple regression model and can be reduced to a sine-wave by synthesizing the function Equation (10). The output form can be taken as the corresponding sinusoidal input.

(8) $${I^{\prime }}_{e}(t)=-3.0\;sin\text{ ​}\left(\frac{2\pi }{T}t\right)\;+\;8.0$$

(9) $${I^{\prime }}_{f}(t)=4.5\;sin\text{ ​}\left(\frac{2\pi }{T}t\right)\;+\;7.0$$

(10) $$f(t)=A\;sin\text{ ​}\left(\frac{2\pi }{T}t+\phi \right)\;+\;c$$

**Table 1:** **Maximum and minimum stimulation amplitude for the six subjects**.

**Subject**	**Biceps**		**Triceps**	
	***I′***_***max***_**(mA)**	***I′***_***min***_**(mA)**	***I′***_***max***_**(mA)**	***I′***_***min***_**(mA)**
A	15.5	6.5	11.5	4.0
B	11.5	2.5	11.0	5.0
C	11.5	7.0	15.0	8.0
D	12.0	4.5	10.0	6.0
E	14.0	6.0	13.0	6.0
F	12.0	3.5	14.0	8.0

In these equations, *A* = $\sqrt{a^{2}+b^{2}}$, sinϕ = *a*/*A*, cosϕ = *b*/*A*, the output amplitude is *A*, the phase lag is ϕ, and the center value of the output sine-wave is *c*.

#### 2.5.2. Estimate of the transfer function

**Figure 5:**
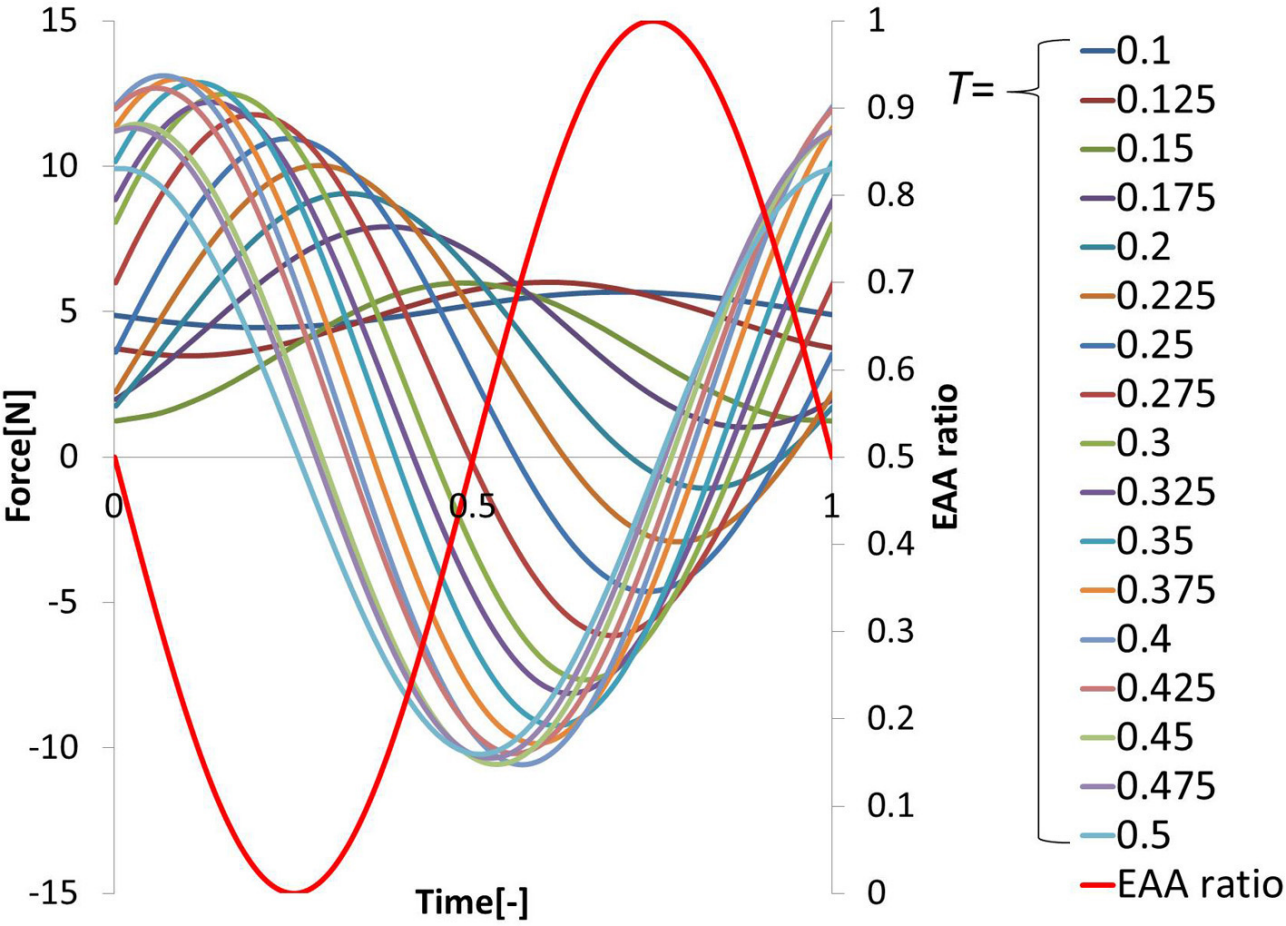
**Input–output data plotted against normalized time (subject B)**.

**Figure 6:**
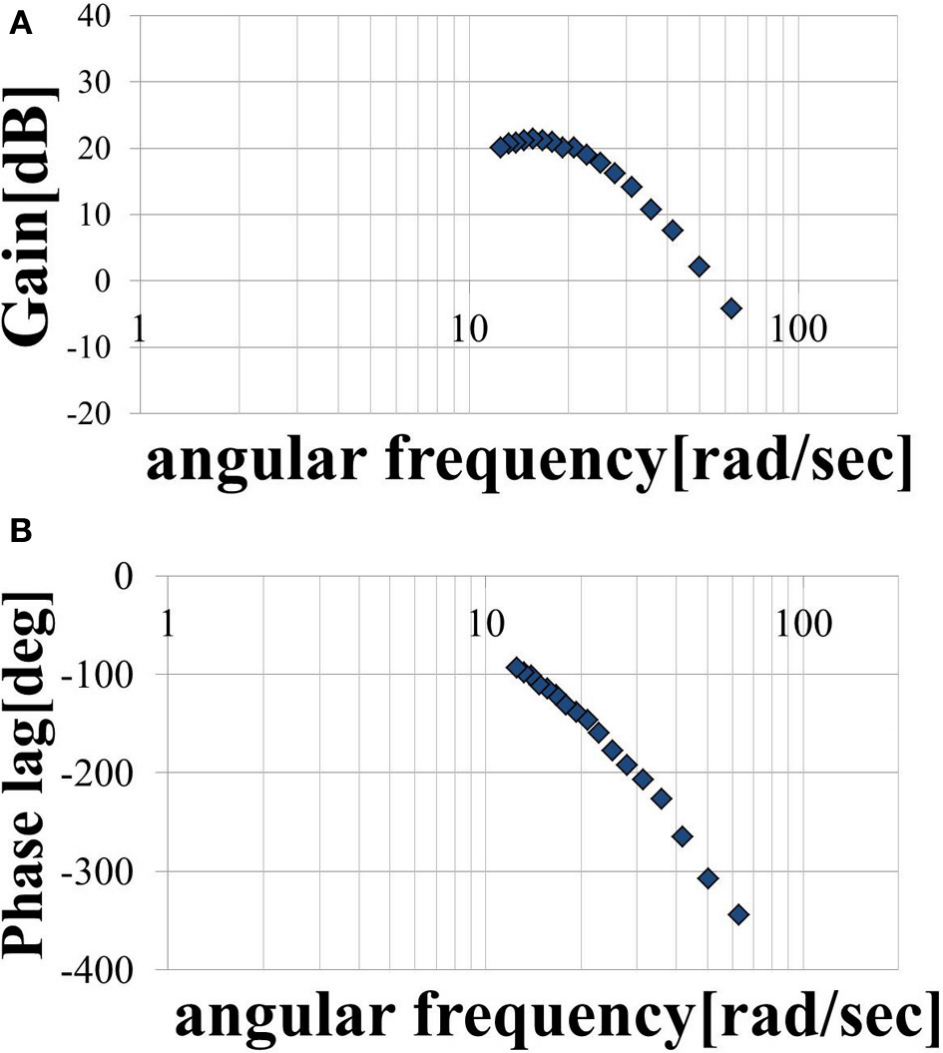
**(A)** Magnitude plot and **(B)** phase plot.

Three trials involving an input of 10 cycles in each period were performed. The period *T* of the sine-wave EAA ratio represented by Equation (7) is incremented by 0.025 (s) in the 0.1–0.5 (s) range. The input was started 0.5 (s) after the start of measurement. After the measurement, the output was approximated as a sine-wave using multiple regression analysis. First, the output values from the three trials were averaged; then, the measured data was divided into 10 cycles of the input sine-wave, and the values of cycles 3–8 were averaged. These cycles represent steady-state behavior. We performed a multiple regression analysis on one cycle of the averaged output, which was approximated by the sine-wave obtained using Equation (10). We normalized the time axis of subject B’s results, shown with the input sine-wave EAA ratio in Figure 5. The results show that the elbow joint system is controlled stably and smoothly via the simultaneous stimulation of multiple muscles based on the EAA ratio when either the hand force switches between positive and negative or the stimulation starts. These situations tend to generate unstable responses when multiple muscles are stimulated at different times. Furthermore, the vibration center of the output is shifted to the positive side (the extension side) when *T* is 0.4 (s) or less, but the amount of shift is approximately 0 (N) when *T* is 0.4 (s) or more. This is due to the difference in the response speeds of the extensor and the flexor, a phenomenon observed only in the high-frequency region of the input. Given that FES is intended to support day-to-day activities, the vibration center shift in the high-frequency input region is not considered to be a serious problem. Therefore, we focus only on the input–output amplitude ratio and the input–output phase difference and attempt to model the input–output relationship of the elbow joint system using a transfer function. Figures 6A,B show the gain diagram and phase diagram for the input and output data shown in Figure 5. The gain is nearly constant in the low-frequency region, and is linearly damped in the high-frequency region, which is typical of an *n*-order delay system. The slope of the high-frequency region, calculated using least squares approximation, is −42.5 (dB/dec) approximation. The gain characteristic is approximated using a second-order delay system. In contrast, the phase diagram shows that the phase has a larger phase lag than the second-order delay system. In this study, this phase delay, which cannot be represented as a second-order lag system, is modeled as a system with dead time.

**Figure 7:**
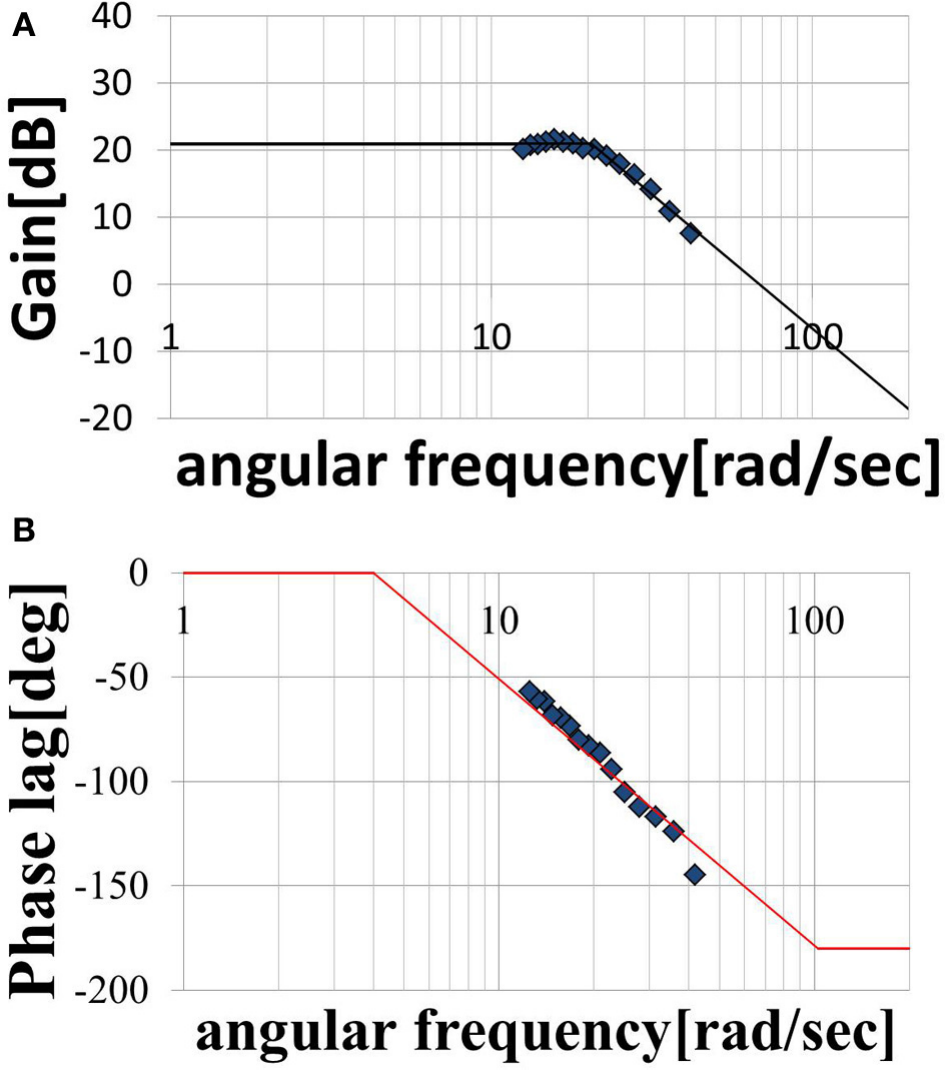
**Modeled (A) magnitude plot and (B) phase plot**.

We assumed that the shape of the transfer function could be represented by Equation (11), where, ω_*n*_ is the natural angular frequency, *K* is a constant, and τ is dead time. We assumed a value of ζ = 1 for the attenuation coefficient. Figures 7A,B show the gain and phase characteristics. These are approximated as a second-order system plus a dead time system, as expressed by Equation (12), and are represented by the broken line. We created similar models using the results obtained for subject A, C, D, E, and F. The results for the six subjects are shown in Table 2. The ω_*n*_, *K*, and τ values for these five subjects differ from those for subject B, but it is understood that all of the subjects’ result can be modeled by transfer functions as a second-order system plus a dead time system. The estimated range of dead times, 0.045–0.100 (s), is consistent with the measured electrical stimulation latency results. We assumed that differences in the parameter values of each individual are related to the ratio of slow-twitch to fast-twitch muscle fibers of an individual and the rate of muscle development. However, in practice, it is possible to determine the optimum parameters values easily for individuals who exhibit some differences. These present modeling method is easy to use and very simple.

(11) $$G(s)=K\;\cdot \;\frac{\omega_{n}^{2}}{s^{2}\;+\;2\zeta \omega_{n}s\;+\;\omega_{n}^{2}}\;\cdot \;e^{-\tau s}$$

(12) $$G(s)=11.22\;\cdot \;\frac{420.25}{s^{2}\;+\;41s\;+\;420.25}\;\cdot \;e^{-0.05s}$$

**Table 2:** **Parameter values for the six subjects**.

**Subject**	**ω**_***n***_ **(rad/s)**	***K***	**τ** **(s)**
A	20.5	11.22	0.050
B	20.5	8.91	0.045
C	31.4	1.73	0.090
D	14.0	1.04	0.100
E	25.1	6.61	0.100
F	18.0	6.96	0.095

## 3. Results and discussion

### 3.1. Verification

In this section, to verify the effectiveness of the model, we present the following three types of hand force control results obtained in the isometric environment. The results for subject B are considered to be verified because the results for all subjects are substantially similar. The six subjects’ multiple coefficients of determination are shown in Table 3.

1. Response to continuously changing input
2. Response to stepwise changing input
3. Interaction with central movement command

**Table 3:** **Multiple coefficients of determination**.

**Subject**	**Experiment (1)**	**Experiment (2)**	**Experiment (3) [during stimulation with +10 (N)]**
A	0.86	0.90	0.95
B	0.99	0.96	0.94
C	0.86	0.71	0.83
D	0.93	0.92	0.77
E	0.97	0.87	0.80
F	0.98	0.75	0.76
Mean	0.93	0.85	0.84
*SD*	0.06	0.10	0.08

#### 3.1.1. Response to continuously changing input

We considered a task in which the direction and magnitude of the hand force change freely. We stimulated the agonist-antagonist muscle pair of the elbow joint using the synthesized EAA ratios of the two types (*T* = 0.3 and *T* = 0.6).

(13) $$r_{E}=0.6\;\left(-0.5sin\frac{2\pi }{0.3}t+0.5\right)\;+\;0.4\;\left(-0.5sin\frac{2\pi }{0.6}t+0.5\right)$$

**Figure 8:**
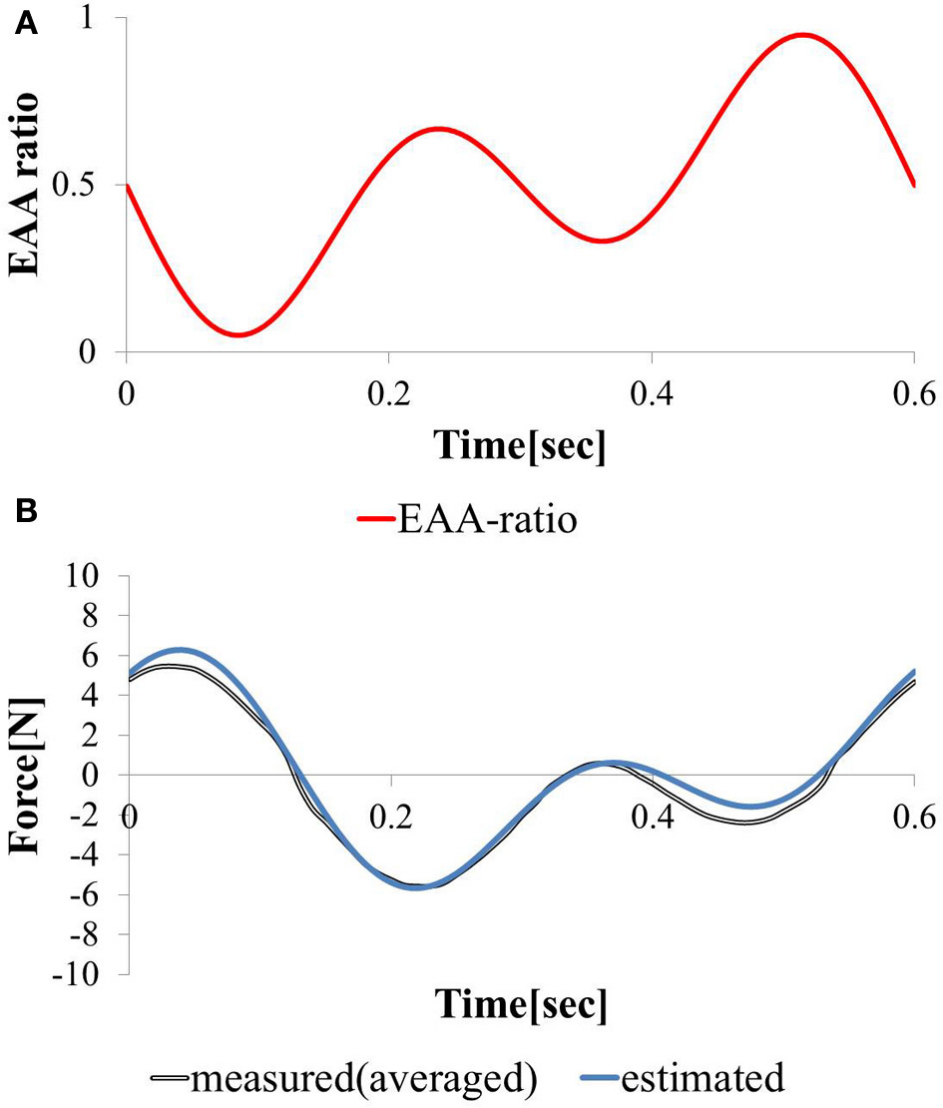
**Responses to continuously varying (A) EAA ratio and (B) estimated and measured**.

The hand force value estimated using model equation (Equation 12) and the measured value of the hand force with the input waveform are shown in Figures 8A,B. Only one input–output cycle [0.6 (s) period] waveform in the steady state are depicted. Because the estimated and measured values of hand force are nearly equal, the validity of the model can be considered confirmed. The EAA ratio for a period of *T* = 0.3 (s) leads to a shift in the vibration center due to the difference between the response speeds of the agonist and antagonist muscles. However, in the input to Equation (13), when combined with the EAA ratio with a period of *T* = 0.6 (s), which is longer than 0.3 (s), there is hardly any shift in the vibration center.

#### 3.1.2. Response to stepwise changing input

**Figure 9:**
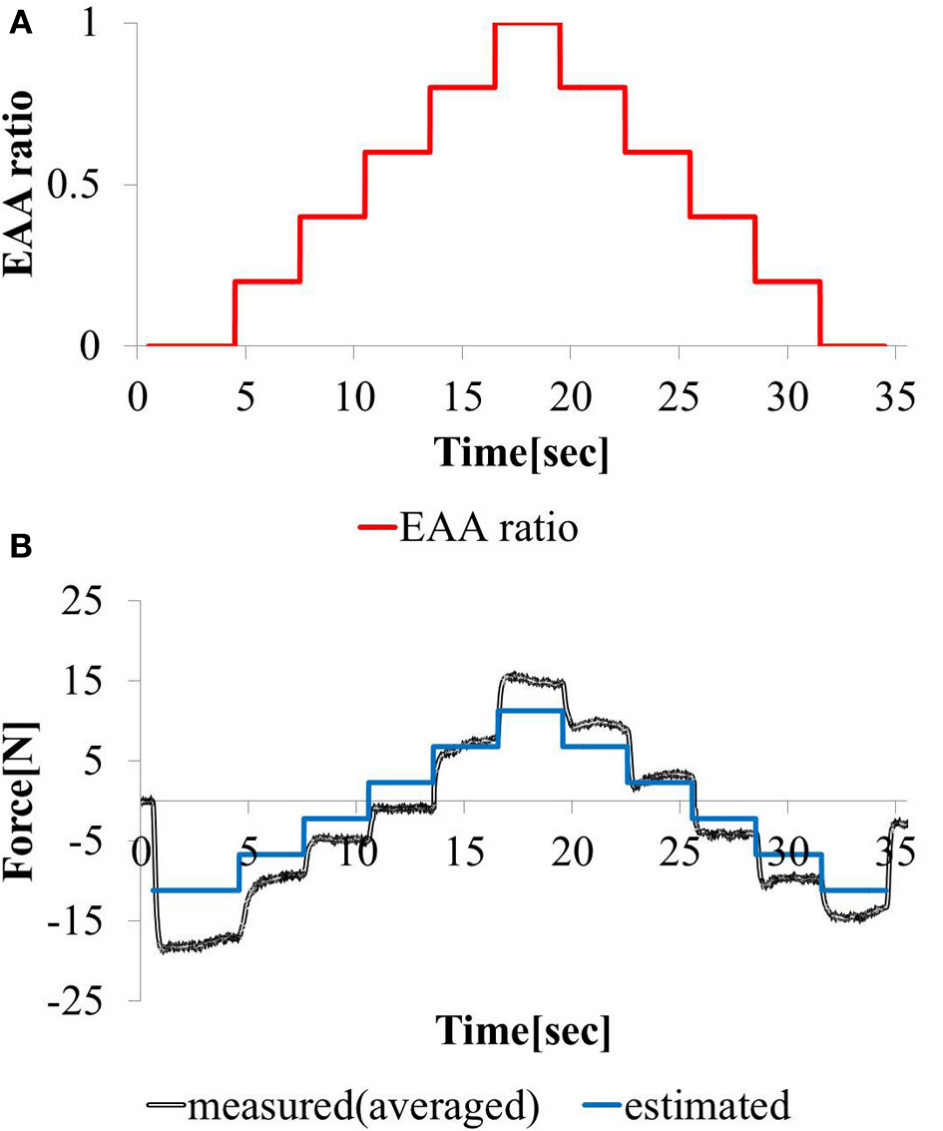
**Responses to stepwise varying (A) EAA ratio and (B) estimated and measured**.

We considered a task with stepwise changes in the hand force magnitude. We increment or decrement the EAA ratio by 0.2 every 3 (s) beginning at *r*_*E*_ = 0. The hand force value estimated using model equation (Equation 12) and the measured value of the hand force with the input waveform are shown in Figures 9A,B. Except when the EAA ratio was near 1 or 0, the difference between the estimated and measured hand force values was 2 (N) or less. The results show that the model can represent steady-state characteristics in a practical manner. When the EAA ratio is near 1 or 0, it is assumed that the extensor or flexor acts alone. Therefore, the model’s estimation error increases.

#### 3.1.3. Interaction with central movement command

In this study, the elbow joint was controlled by the EAA ratio, which is considered an equilibrium point, as shown in Figure 4. It was assumed that the equilibrium point is operated by a scheme representing the sum of the FES commands given based on the external and motion commands from the central nervous system. To validate this theory, we performed an experiment in which electrical stimulation was provided in a state in which the subject was generating hand force intentionally. The subject was confirmed that he maintained a positive or negative hand force of approximately 10 (N) without feedback. In addition, our input pattern given by Equation (14) was limited to only two cycles [for 1 (s)]. We repeated this pattern three times at intervals of 2.0 (s).

(14) $$r_{E}=-0.5\;sin\text{ ​}\left(\frac{2\pi }{0.5}(t-a)\right)\;+\;0.5$$

**Figure 10:**
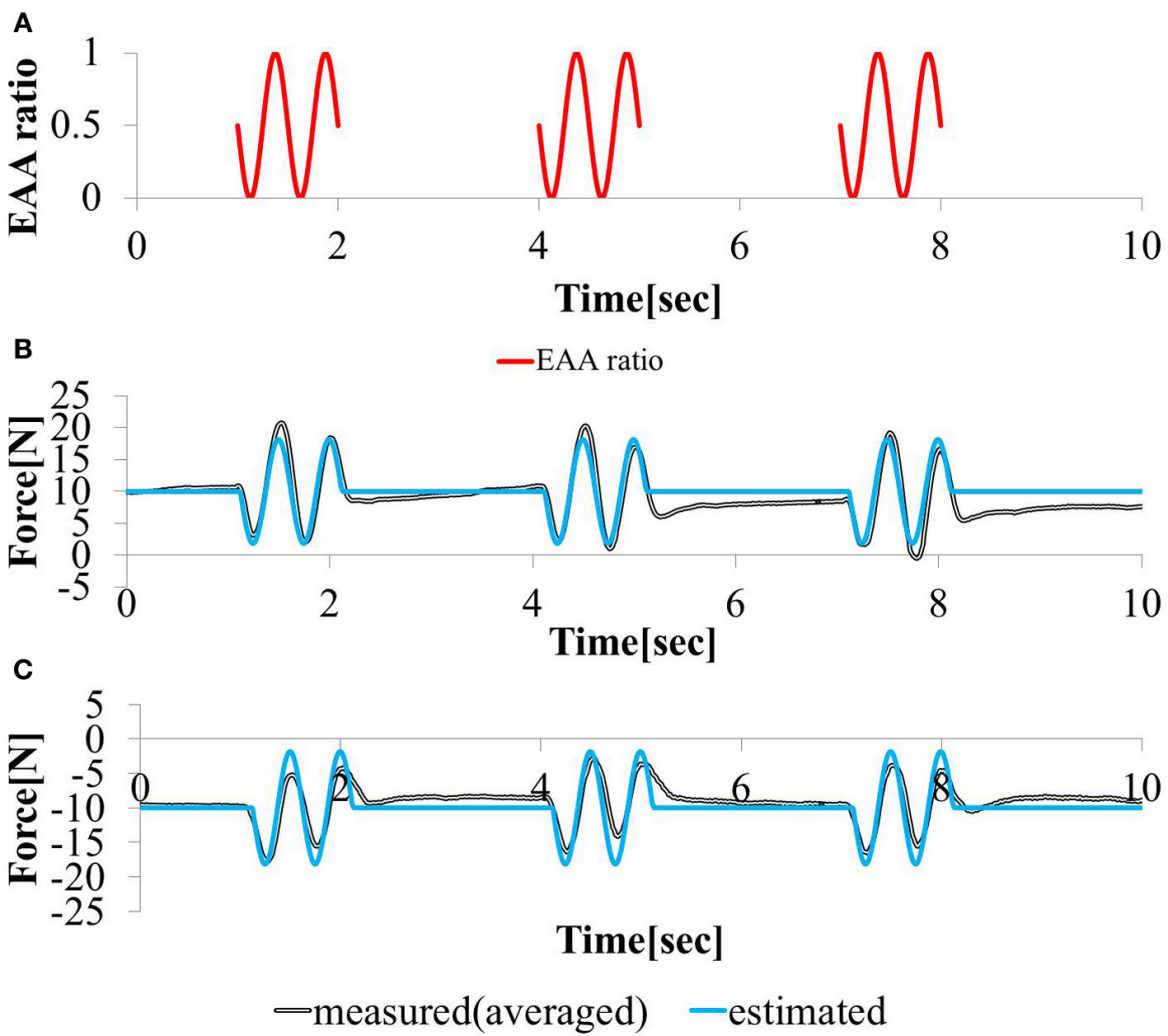
**Responses to sinusoidal (A) EAA ratio in (B) positive (C) negative 10 (N) force exertion task**.

The input stimulation was applied only when *a* ≤ *t* ≤ *a* + 1 (s) with *a* = {1, 4, 7}. The hand force was estimated using model equation (Equation 12), The measured value of the hand force and the input waveform are shown in Figures 10A–C. We stimulated the agonist-antagonist muscle pair in the elbow joint based on the EAA ratio given by Equation (14) while the subject was generating approximately +10 (N) of hand force (in the elbow extension direction). The results are shown in Figure 10B. In addition, we stimulated the agonist-antagonist muscle pair of the elbow joint based on the EAA ratio given by Equation (14) while the subject was generating approximately −10 (N) of hand force (in the elbow flexion direction). The results are shown in Figure 10C. In both cases, the results show that the hand force was maintained at a positive or negative value of approximately 10 (N) in the section without electrical stimulation. The hand force varied in the section with electrical stimulation, and the magnitude of these changes were close to the hand force value obtained using Equation (12). These results indicate that FES stimulation can be useful in supporting daily human motions. That is, day-to-day human tasks, we can design the necessary movement support system based on FES, using motion commands from the central nervous system.

## 4. Conclusions

In this study, muscle co-contraction was employed in FES. We focused on the agonist-antagonist muscle pair that drives the elbow joints. We proposed an electrical stimulation method that stimulates units of agonist-antagonist muscle pairs. The effectiveness of the proposed method was validated through experiments requiring control of the hand force of a single elbow joint with activation of one agonist-antagonist muscle pair in an isometric environment using six subjects. Based on the results obtained from performing simultaneous stimulation of multiple muscles based on the EAA ratio, we can draw the following conclusions.

- Using the electrical stimulation method proposed as an open-loop control in this paper, stable and smooth control can be more easily achieved than with other methods (Kurosawa et al., 2005), especially when the sign of the hand force switches.
- We can define the elbow joint as a system with an input (the EAA ratio corresponding to the target value of the joint equilibrium point) and an output (the hand force). The system can be modeled as a cascaded second-order system with dead time.
- Using the model developed in this study, the hand force that will be generated by a predetermined electrical stimulation pattern can be accurately estimated.

These findings indicate that our proposed method is an effective solution to the problem of redundancy in an agonist-antagonistic drive system and non-linearity between stimulus current values and muscle force/length. We indicated the possibility that high-speed, highly accurate hand force control can be achieved using this model as an inverse system. This model can also be used for tasks involving joint motion, if this model is applied as a rigid body link model (input:joint torque, output:joint angle).

The results of the experiment in which electrical stimulation was conducted together with the conscious application of hand force demonstrate that FES can be used to design a system to provide the necessary movement support for daily human tasks using motion commands from the central nervous system.

It is necessary to ensure that stimulation patterns can be adjusted according to the requirements of the FES application to a variety of tasks. Previous FES studies might have inadvertently neglected the regulation of additional properties involved in coordinating various muscles such as joint stiffness or methods of dealing with muscle redundancy (Jarc et al., 2013). Our proposed method offers the following advantages:

- Independent control of the joint equilibrium point and joint stiffness,
- Accurate realization of isometric tasks through EAA ratio-based equilibrium-point control with EAA activity *a*_*E*_ = 1, and
- Easy extension of the proposed method to the muscle synergy control method, which can be applied to controlling various muscles simultaneously.

In this study, the environment was limited to being isometric, with the moving joint limited to being only an elbow joint and fatigue is excluded. We normalized the FES intensity to a level at which the subject did not feel pain. In our future research, we plan to normalize the FES intensity at a level at which the force is balanced. In the future, we will apply the proposed method to tasks with joint motion, multiple joints, and tasks performed for long periods of time to further validate the effectiveness of the method.

### Conflict of interest statement

The authors declare that the research was conducted in the absence of any commercial or financial relationships that could be construed as a potential conflict of interest.
